# Left Main Coronary Artery (LMCA) Iatrogenic Dissection: A Case Report of a Catastrophic Complication During Primary Percutaneous Coronary Intervention (PCI)

**DOI:** 10.7759/cureus.100173

**Published:** 2025-12-27

**Authors:** Youssef Daoudi, Oumaima T Taoussi, Ghita Bennis, Hibat Allah Kamri, Fatima-Zahra Merzouk

**Affiliations:** 1 Cardiology, Cheikh Khalifa International University Hospital, Casablanca, MAR; 2 Cardiology, Mohammed VI University of Sciences and Health, Casablanca, MAR

**Keywords:** academic cardiology, coronary artery angiography, coronary intervention, coronary ischemia, iatrogenic coronary dissection

## Abstract

Catheter-induced coronary artery dissection is a rare and serious complication that may occur during coronary angiography or angioplasty procedures. Its timely recognition and appropriate management are critical to prevent severe consequences such as myocardial infarction or sudden cardiac death. The treatment strategy depends on the extent and location of the dissection, with revascularization often required to restore adequate coronary blood flow. Various approaches, including percutaneous intervention, may be employed based on the clinical scenario. In this report, we describe a case of successful recanalization following an iatrogenic coronary dissection, emphasizing the importance of early intervention and individualized management.

## Introduction

Iatrogenic catheter-induced coronary artery dissection is a rare but serious complication of coronary angiography and percutaneous coronary intervention (PCI), with an incidence of less than 0.1% [1]. It can lead to severe outcomes such as myocardial infarction, hemodynamic instability, or death, particularly when the left main coronary artery (LMCA) is involved. Most cases occur during interventions on the right coronary artery (RCA), followed by the left anterior descending artery (LAD) and LMCA [2]. The mechanisms include mechanical trauma from the catheter or forceful contrast injection, especially in patients with vulnerable vessel walls. Management depends on the extent and location of the dissection, ranging from stenting to emergency surgery. This case highlights a rare iatrogenic LMCA dissection during primary PCI and its successful percutaneous management.

## Case presentation

**Figure 1:**
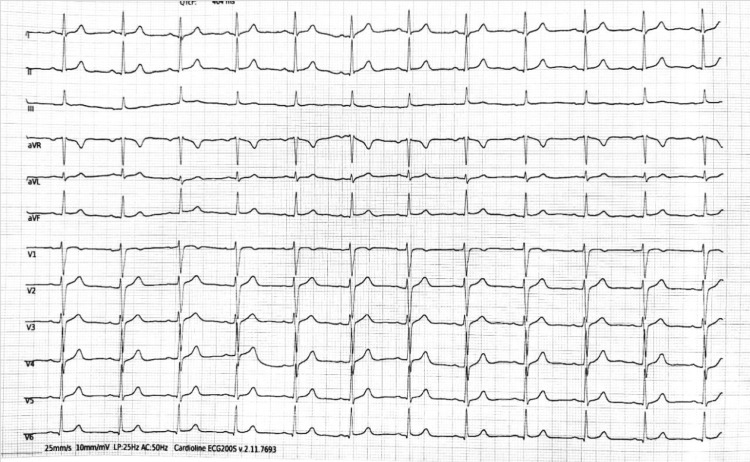
ECG of the patient

A 66-year-old active smoker had been experiencing effort-induced angina (Canadian Cardiovascular Society or CCS class II) for the past three months. Clinical examination and ECG demonstrated poor R-wave progression in leads V1-V3 (Figure 1). 

Due to intermediate pretest probability of coronary artery disease, the patient underwent an exercise stress test, which was positive for ischemia based on horizontal ST depressions in leads V4-V6 during peak exercise. Based on these findings, the patient was discharged, and a coronary angiography was scheduled on an outpatient basis.

While awaiting coronary angiography, the patient experienced typical anginal chest pain, which he neglected. Clinical examination remained normal, but the ECG revealed a consumed myocardial infarction with elevated troponin levels.

**Figure 2:**
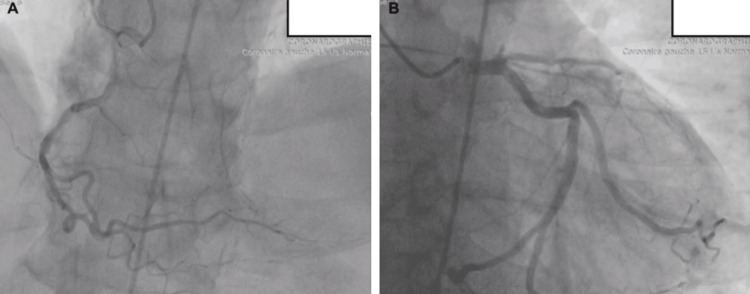
Coronary angiography of the patient showing proximal LAD occlusion A: Right coronary artery; B: Left coronary artery with proximal left anterior descending artery (LAD) occlusion.

Echocardiography showed evidence of ischemic heart disease, with apical akinesia and affected anterior and anterolateral walls. Coronary angiography confirmed proximal LAD occlusion (Figure 2).

**Figure 3:**
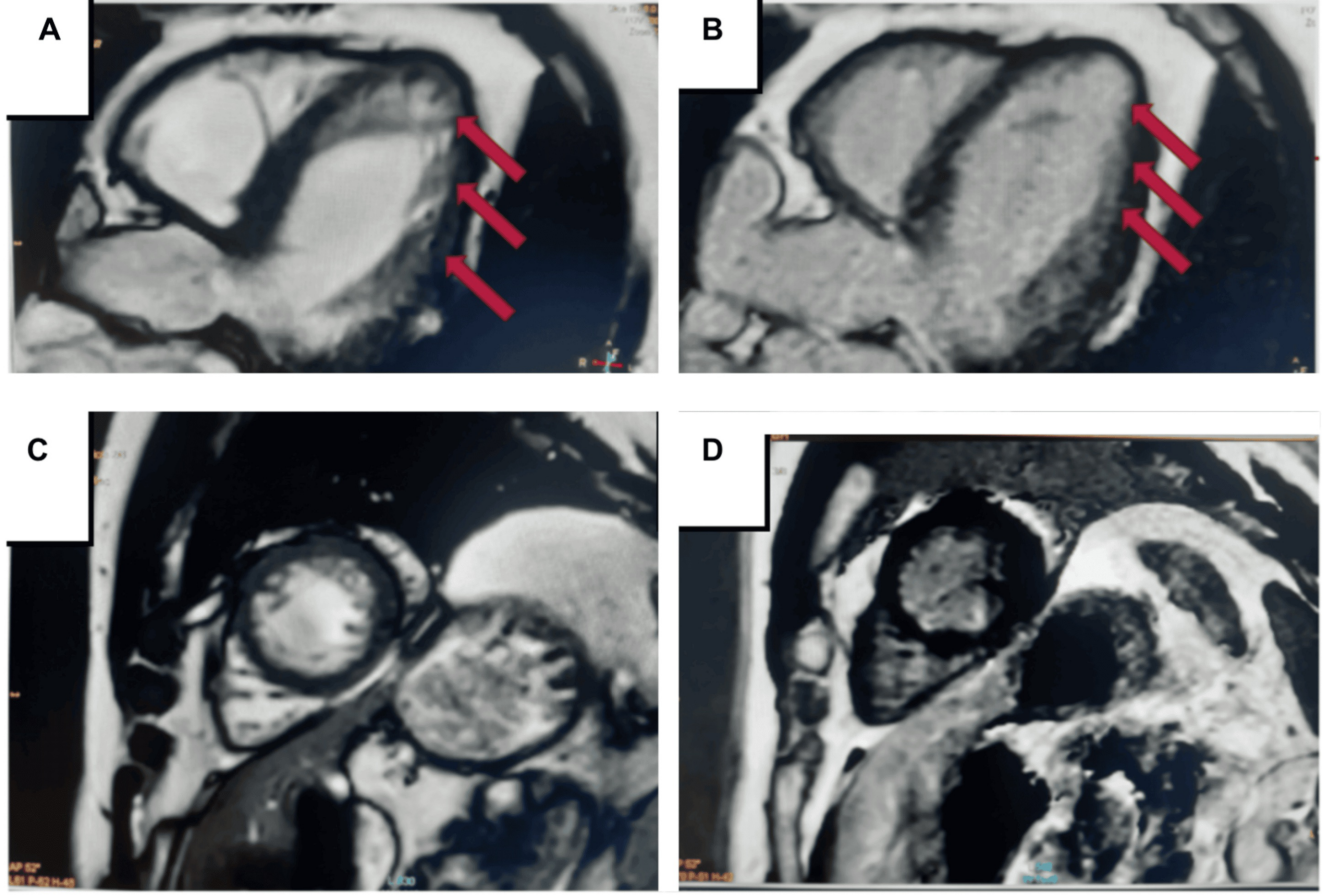
MRI images Ischemic heart disease with moderate systolic dysfunction of the left ventricle, left ventricular ejection fraction (LVEF) 50%. Red arrows indicate areas of late gadolinium enhancement; preserved myocardial viability is evident in the left anterior descending artery (LAD) territory. No endocardial thrombus was observed. No endocardial thrombus observed. A,B: 4-chamber view; C,D: Short-axis view.

Revascularization was indicated because MRI viability testing demonstrated preserved myocardial viability in the LAD territory (Figure 3).

**Figure 4:**
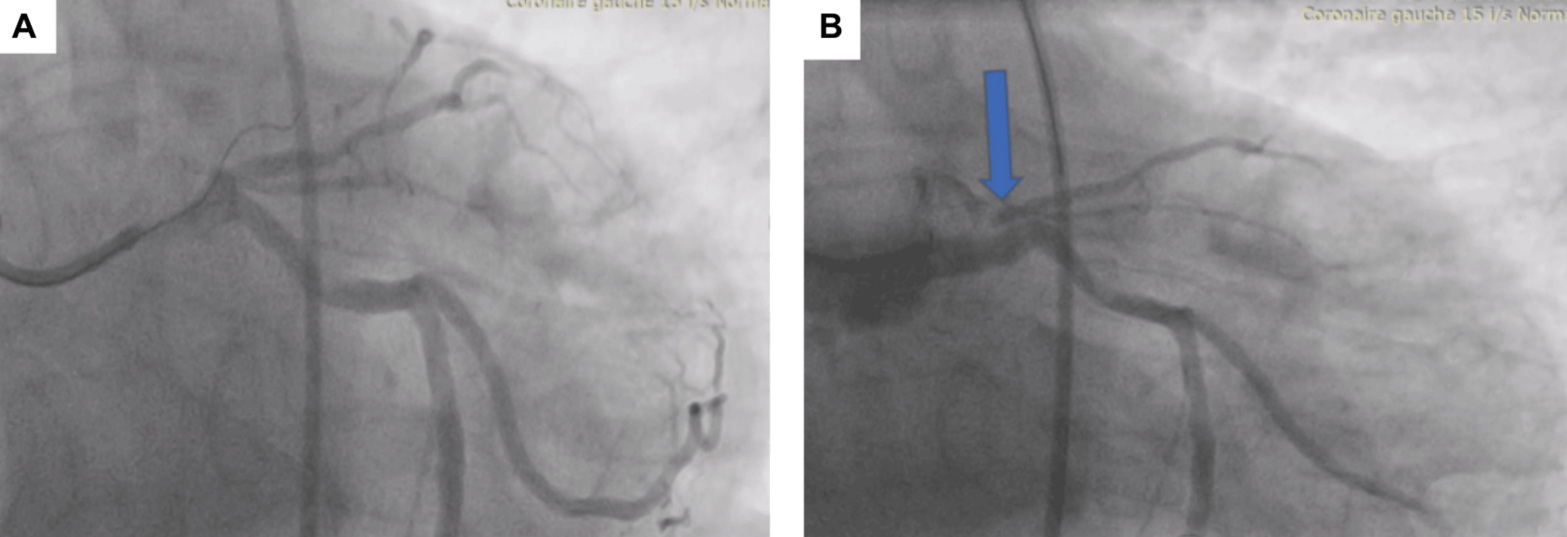
A coronary dissection was suspected The blue arrow in the figure shows the contrast agent outside of the left anterior descending artery (LAD). A: Wire crossing the LAD occlusion; B: Coronary artery dissection with contrast extravasation outside the LAD on angiography.

During PCI, after passing the guidewire through the LAD stenosis, coronary angiography revealed the contrast agent stranded in the proximal LAD and spreading outside the aortic wall. At this time, the patient felt chest pain, had anterior ST elevation, and developed hemodynamic instability. A coronary dissection of the left main coronary artery was confirmed (Figure 4).

**Figure 5:**
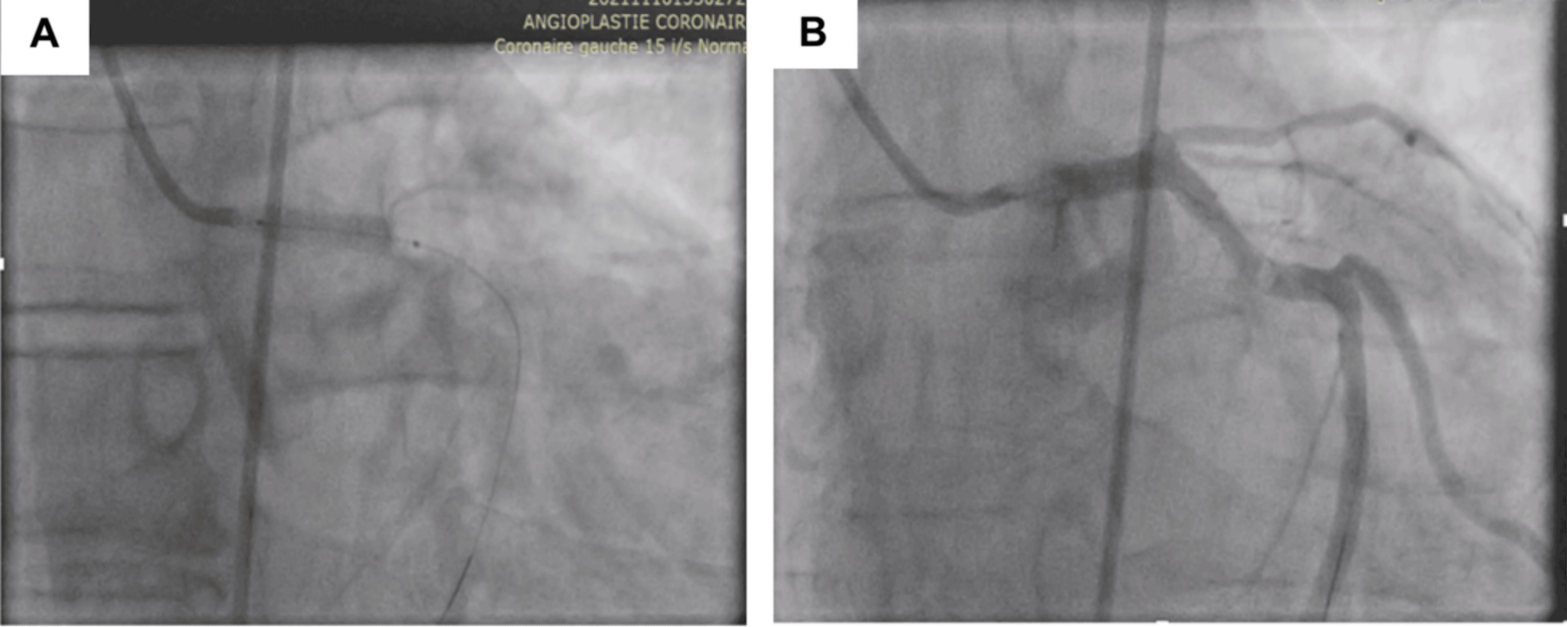
Coronary stenting of the LCA A: Coronary stenting of the LCA (left coronary artery); B: No contrast agent extravasation after the stenting of the LCA.

The patient went into cardiac arrest, and immediate cardiac resuscitation was initiated. The patient was intubated and placed on vasopressive drugs. A guidewire was inserted into the circumflex artery, and a stent was implanted in the LCA to seal the dissection entry site (Figure 5).

**Figure 6:**
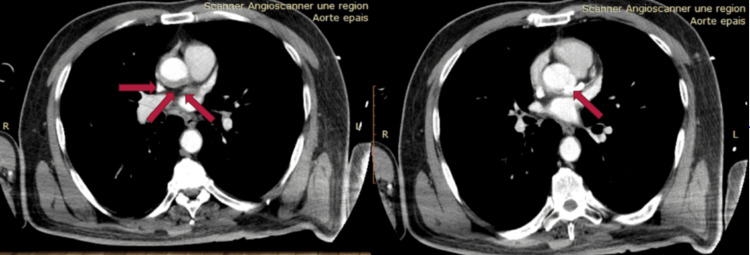
Aortic computed tomography angiography The red arrow shows circumferential transmural hematoma at the sino-tubular junction to be monitored. The stent is in place and there is no aortic dissection.

In the following days, the patient was weaned off vasopressive drugs and extubated. Echocardiography revealed apical, anterior, and anterolateral hypokinesia of the left ventricle with an ejection fraction of 45%, and no pericardial effusion. The patient was in good general condition and symptom-free from angina. A CT scan performed five days later showed a circumferential transmural hematoma at the sino-tubular, with the stent in place, and no aortic dissection (Figure 6).

Ten days later, the patient was discharged with a recommendation for dual antiplatelet therapy (aspirin and clopidogrel) for 12 months. Follow-up coronary angiography after six weeks showed successful PCI outcomes for the LMCA.

## Discussion

Catheter-induced coronary artery dissection is a serious complication of coronary catheterization. Most cases occur during interventions on the RCA, with the LAD being the next most commonly involved, the LMCA and the left circumflex artery (LCX) [3]. Clinical presentations range from a stable, localized, and asymptomatic tear to a more severe form involving an extensive intramural hematoma. In our case, the hematoma resulted in arterial lumen compression, obstructing blood flow and potentially leading to acute coronary syndrome, myocardial infarction, or even sudden cardiac death [4].

The dissection can result from direct aggressive manipulation of the guiding catheter or indirectly from forceful contrast medium injection into the coronary ostium, especially in fragile vessel walls [5]. In our case, we believe the dissection was primarily caused by forceful handling of the guiding catheter, rather than by the angioplasty guidewire at the intimal level.

Cardiac imaging plays a crucial role in diagnosing the type of dissection, based on angiographic appearances [6]. In our case, the patient had a type C coronary dissection. A coronary-aortic CT scan remains the gold standard for determining the extent of the coronary dissection and identifying any involvement of the aorta [7].

In most cases, the dissection does not progress; stenting at the entry point of the dissection is effective for limited dissections. Immediate stenting has proven highly successful and should be attempted whenever possible. Surgical intervention should be reserved for complex cases involving unstable patients, particularly when the dissection extends into the aorta, as these cases carry a high mortality rate [8].

## Conclusions

This case underscores the potentially catastrophic nature of iatrogenic LMCA dissection during PCI, emphasizing the importance of early recognition and decisive management. It highlights the need for meticulous catheter manipulation, particularly in high-risk anatomical regions such as the LMCA. The successful outcome in this patient was made possible through immediate hemodynamic support, prompt sealing of the dissection entry point with a stent, and close post-procedural monitoring. Clinicians must remain vigilant for signs of dissection during angiography and be prepared to act swiftly to prevent progression to myocardial infarction or cardiovascular collapse. This case reinforces that, when managed promptly and effectively, even severe iatrogenic complications can have favorable outcomes.
